# The Incidence and Types of Medication Errors in Patients Receiving Antiretroviral Therapy in Resource-Constrained Settings

**DOI:** 10.1371/journal.pone.0087338

**Published:** 2014-01-28

**Authors:** Kenneth Anene Agu, Dorothy Oqua, Zainab Adeyanju, Muhammadu Alfa Isah, Afusat Adesina, Samuel I. Ohiaeri, Pollock N. Ali, Nnenna Ekechukwu, Augustine Adah Akpakwu, Tindak Sani, Idoko Onuche Omeh, Rosalyn C. King, Anthony K. Wutoh

**Affiliations:** 1 Howard University Pharmacists And Continuing Education (PACE) Center, Abuja, Nigeria; 2 Howard University College of Pharmacy, PACE Center, Washington DC, United States of America; Istituto Superiore di Sanità, Italy

## Abstract

**Purpose:**

This study assessed the incidence and types of medication errors, interventions and outcomes in patients on antiretroviral therapy (ART) in selected HIV treatment centres in Nigeria.

**Methods:**

Of 69 health facilities that had program for active screening of medication errors, 14 were randomly selected for prospective cohort assessment. All patients who filled/refilled their antiretroviral medications between February 2009 and March 2011 were screened for medication errors using study-specific pharmaceutical care daily worksheet (PCDW). All potential or actual medication errors identified, interventions provided and the outcomes were documented in the PCDW. Interventions included pharmaceutical care in HIV training for pharmacists amongst others. Chi-square was used for inferential statistics and P<0.05 indicated statistical significance.

**Results:**

Of 6,882 participants, 67.0% were female and 93.5% were aged ≥15years old. The participants had 110,070 medications filling/refilling visits, average (±SD) of 16.0 (±0.3) visits per patient over the observation period. Patients were followed up for 9172.5 person-years. The number of drug items dispensed to participants was 305,584, average of 2.8 (±0.1) drug items per patient. The incidence rate of medication errors was 40.5 per 100 person-years. The occurrence of medication errors was not associated with participants’ sex and age (*P*>0.05). The major medications errors identified were 26.4% incorrect ART regimens prescribed; 19.8% potential drug-drug interaction or contraindication present; and 16.6% duration and/or frequency of medication inappropriate. Interventions provided included 67.1% cases of prescriber contacted to clarify/resolve errors and 14.7% cases of patient counselling and education; 97.4% of potential/actual medication error(s) were resolved.

**Conclusion:**

The incidence rate of medication errors was somewhat high; and majority of identified errors were related to prescription of incorrect ART regimens and potential drug-drug interactions; the prescriber was contacted and the errors were resolved in majority of cases. Active screening for medication errors is feasible in resource-limited settings following a capacity building intervention.

## Introduction

A medication error is defined "as any preventable event that may cause or lead to inappropriate medication use or patient harm while the medication is in the control of the health care professional, patient, or consumer [1]. Such events may be related to professional practice, health care products, procedures, and systems, including prescribing, order communication, product labelling, packaging, and nomenclature, compounding, dispensing, distribution, administration, education, monitoring and use" [1]. Medication errors are often times a concern in chronically ill patients most likely because of the increased likelihood for poly-pharmacy and longer durations of therapy [2], [3], [4]. In patients infected with Human Immunodeficiency Virus (HIV), the risk of a medication error occurring is higher as patients continue the lifelong antiretroviral therapy (ART). These patients may also require treatment for co-morbidities like diabetes, renal and cardiovascular diseases which further increase this risk [5].

Some medication errors that are most frequently occurring in an HIV/AIDS clinic setting include non-recommended drug-drug combinations, incorrect dosing schedules and incorrect dosages [6], [7]. These challenges with medication use might result from the prescriber, the pharmacist or the patient and may tend to be more in HIV clinic settings with high patient burden and with limited HIV specialist care [7], [8], [9]. In both urban and rural settings in communities within Nigeria, this is often times the typical scenario. The HIV burden is high in several communities and health facilities are ill equipped with the specialist health workforce to cope with the rising number of patients attending clinics and taking antiretroviral medications [10], [11]. Opportunities for medication reconciliation to prevent medication errors are very few and almost non-existent in these clinics as patient to clinician or patient to pharmacist contact times are very short or even sometimes almost unavailable.

There is limited information on the incidence and types of medication errors as well as the interventions provided in clinics providing HIV care and treatment in Nigeria. This study seeks to elucidate the incidence and types of medication errors, the interventions and outcomes of medication errors in patients receiving antiretroviral therapy in selected hospitals in Nigeria.

## Methods

### Study design

The study was a prospective cohort assessment of patients receiving antiretroviral therapy (ART) between February 2009 and March 2011 in outpatient pharmacy. The patients were screened for medication errors based on the provisions of the national HIV care and treatment guideline [12].

### Setting

The study was conducted in 14 secondary public hospitals providing free antiretroviral therapy to patients who were eligible to commence treatment based on the national HIV care and treatment guideline [12]. The comprehensive HIV care and treatment programme in these health facilities was implemented by Global HIV/AIDS Initiative Nigeria (GHAIN) project with funding support from President Emergency Plan for AIDS Relief (PEPFAR) through United States Agency for International Development (USAID). All patients who tested positive for HIV were assessed for ART eligibility and only those who were eligible based on the national HIV care and treatment guideline [12] were commenced on ART. All ART-ineligible patients were enrolled into pre-ART register and were provided pre-ART care in accordance with the provisions of the national HIV care and treatment guideline [12]. All ART patients who came to the pharmacy for filling/refilling of their antiretroviral medications were routinely screened for medication errors using pharmaceutical care daily worksheet (PCDW). All potential or actual medication errors identified and the interventions provided towards resolving them were documented in the PCDW.

### Selection criteria

All patients who started antiretroviral therapy, filled/refilled their medications and were screened for medication errors between February 2009 and March 2011 were eligible to be included in the study. All GHAIN-supported health facilities that had pharmaceutical care directed active screening for drug therapy problems and had documentation of medication errors identified and the interventions provided were eligible to be included. Patients and health facilities that did not meet these criteria were not included.

### Population/Sample

Out of the 69 GHAIN-supported health facilities that had pharmaceutical care directed active screening for drug therapy problems, 14 of them were selected using simple random sampling techniques. All patients who started antiretroviral therapy and filled/refilled their medications between February 2009 and March 2011 were selected for the study.

### Interventions

The study interventions included training of pharmacists on pharmaceutical care in HIV followed by a hands-on pharmacy best practices training. The training was aimed at improving their knowledge and skills to identify and resolve medication errors in HIV treatment setting based on the provisions of the national HIV treatment guidelines [12]. The pharmaceutical care daily worksheet (PCDW) was designed and deployed to the health facilities for active screening of patients receiving antiretroviral drugs for medication errors and suspected adverse drug reactions at each medication fill/refill visits. Monthly technical assistance was provided (as necessary) to the pharmacists in the health facilities by a team of monitoring and evaluation pharmacists from Howard University Pharmacists And Continuing Education (HU PACE) Center, a core partner in the GHAIN project. No monetary incentive was provided to the health workers involved in the program. Other health providers (medical doctors, pharmacists and nurses) were also trained on the use of antiretroviral drugs for HIV prevention and treatment based on the provisions of the national HIV treatment guidelines [12].

### Outcomes variables

The occurrence of medication errors disaggregated by type during the study period was the main outcome variable. This variable was defined as any preventable event that may cause or lead to inappropriate medication use or patient harm while the medication is in the control of the health care professional, patient, or consumer.

### Ethical consideration

The ethical approval for this study was obtained from National Health Research Ethics Committee (NHREC) Abuja, Nigeria. Informed consent was obtained from the patients and their identifiers were excluded during the data analysis to maintain confidentiality.

### Data collection

A study-specific pharmaceutical care daily worksheet (PCDW) was used for active screening of patients for medication errors at each medication filling/refilling visits. PCDW included sections on patients' demographics, ART regimens, and list of potential/actual medication errors, interventions and outcomes. It also included section for documentation of suspected adverse drug reactions in these patients. Patients refill their antiretroviral medications on bimonthly basis and they are screened for medication error(s) at each visit. All medication errors identified during the screening, the interventions provided and the outcomes of such interventions were routinely documented in the PCDW by the pharmacist. The data on the number of ART patients served in the pharmacy were obtained from the PCDW and was validated with the pharmacy daily worksheet. The pharmacy daily worksheet is a national register for documentation of the quantities of all drugs dispensed to patients. This register also has sections on patients' demographics.

### Data analysis

PASW statistics® version 18 was used for data analysis. Descriptive statistics which included frequency distribution of key items was conducted. Bivariate analysis was conducted to describe the relationship between variables (age and gender). The person-time (years) at risk during the follow up period was calculated as “the sum of each individual's time at risk (i.e. sum of the length of time they were followed up in the study)”. The incidence rate of medication errors was calculated as “the number of ART patients reporting events (medication errors) between February 2009 and March 2011 divided by the amount of person-time at risk during the follow up period (between February 2009 and March 2011). Patients who were noted as having transferred out, dead, loss to follow up or stopped treatment or contributed to the analysis if they had reported medication error(s) before been declared no longer a patient of the health facility. The incidence rate of medication errors was expressed as the number of patients with at least one occurrence of the event (medication error) per 100 person-years. The percentage of patients who had medication error(s) and were provided interventions during the observation period was calculated as “the number of patients who were provided medication error(s) interventions during the observation period divided by the number of patients who were documented to have medication error(s) during the observation period x 100”. The number of drug items dispensed was calculated as the sum of all drug items dispensed to the patients in the pharmacy order form (prescription sheet). Chi-square statistic was used to test the association between reported medication errors and key variables (age and sex). All reported *P* values were 2 - tailed and *P*< 0.05 indicated statistical significance.

## Results

Of the 6,882 patients receiving antiretroviral medicines in the study sites during the observation period, 67.0% were female and 93.5% were aged ≥15 years old (Table 1). Patients were followed up for 9172.5 person-years. These patients had 110,070 medications filling/refilling visits, an average (±SD) of 16.0 (±0.3) visits per patient over the observation period. The number of drug items dispensed to the patients during these visits was 305,584; an average of 2.8 (±0.1) drug items per patient giving indication of the extent of polypharmacy or concomitant medicines use. All patients received different antiretroviral therapy regimens as recommended in the Nigerian national treatment guidelines [12]; however, the patterns of regimens use were not tracked for analysis in this study.

**Table 1:** Socio-demographic characteristics of study participants; N  =  6,882.

Characteristics	Frequency	Percentage (%)
Sex		
Male	2,270	33.0
Female	4,612	67.0
**Age (years)**		
<15	446	6.5
≥15	6,436	93.5

All patients who filled/refilled their medication in the pharmacy during the observation period were screened for medication errors. Of these participants, 65.9% of females and 93.4% of those aged ≥15 years old had medication errors. Of the participants who had medication errors, 98.3% of patients aged ≥15 years old received interventions for the medication errors compared to 62.2% for those aged <15 years old. The overall incidence rate of medication errors in the HIV treatment setting was 40.5 per 100 person-years (Table 2). The incidence rate of medication error among patients aged ≥15 years was greater than that for patients aged <15 years (Table 2); and the difference was not statistically significant (*P*  =  0.281). The occurrence of medication errors was not associated with sex (*P*  = 0.094).

**Table 2:** Medication errors monitoring indicators disaggregated by age and sex of the patients; values in parenthesis are percentages.

Indicators	Sex	Frequency (%)		Total, N (%)
		<15 Years	≥15 Years	
Number of patients' visit to the pharmacy for medication pick-up during the observation period.	Male	5410 (4.9)	31819 (28.9)	37229 (33.8)
	Female	5428 (4.9)	67413 (61.2)	72841 (66.2)
	**Total**	**10838 (9.8)**	**99232 (90.2)**	**110070 (100.0)**
Number of patients who visited the pharmacy for medication pick-up and were screened for medication error during the observation period*	Male	5410 (4.9)	31819 (28.9)	37229 (33.8)
	Female	5428 (4.9)	67413 (61.2)	72841 (66.2)
	**Total**	**10838 (9.8)**	**99232 (90.2)**	**110070 (100.0)**
Number of patients who were screened and documented to have medication error(s) during the observation period.	Male	129 (3.5)	1137 (30.6)	1266 (34.1)
	Female	117 (3.1)	2332 (62.8)	2449 (65.9)
	**Total**	**246 (6.6)**	**3469 (93.4)**	**3715 (100.0)**
Number of patients who were provided medication error(s) interventions during the observation period.	Male	76 (2.1)	1133 (31.8)	1209 (33.9)
	Female	77 (2.2)	2278 (63.9)	2355 (66.1)
	**Total**	**153 (4.3)**	**3411 (95.7)**	**3564 (100.0)**
**Indicators**	**Sex**	**Percentage**		**Total (%)**
		**<15 Years**	**≥15 Years**	
Percentage of patients who had medication error(s) and were provided interventions during the observation period.	Male	58.9	99.6	95.5
	Female	65.8	97.7	96.2
	**Total**	**62.2**	**98.3**	**95.9**
**Indicators**	**Sex**	**Incidence rate (per 100 person-years)**		
		**<15 Years**	**≥15 Years**	**Total**
Incidence rates of medication errors per 100 person-years disaggregated by age and sex.	Male	28.6	42.9	40.8
	Female	25.9	41.5	40.3
	**Total**	27.2	42.0	40.5

* Some patients had repeated screening for medication error at every medication refill visits.

The major medications errors identified were 26.4% incorrect antiretroviral drugs combinations/regimens prescribed; 19.8% possible drug-drug interaction or contraindication present; and 16.6% duration and/or frequency of medication inappropriate (Table 3). The interventions provided by the pharmacists included 67.1% cases of prescriber or other health workers contacted to clarify or resolve the errors; and in 14.7% of cases patient counselling and education were provided. The potential/actual medication error(s) were resolved in 97.4% of the cases (Table 3).

**Table 3:** Distribution of medication errors identified, interventions taken and the outcomes in patients receiving antiretroviral therapy (ART); N  =  3715*.

Medication errors identified	Frequency	Percentage
Antiretroviral therapy (ART) ineligible client commencing ART	131	3.4
Incorrect dose (Low dose or high dose) prescribed	307	7.9
Incorrect antiretroviral drugs combinations/regimens prescribed	1028	26.4
No drug for the medical problem	318	8.2
No valid indication for the drug	229	5.9
Possible drug-drug interaction or contraindication present	772	19.8
Prescription order with incomplete prescriber/client details	463	11.9
Duration and/or frequency of medication inappropriate	647	16.6
**Total**	**3895**	**100.0**
**Medication error(s) interventions provided**		
Prescriber or other health workers contacted to clarify/resolve error(s)	2653	67.1
Drug therapy initiated/changed by prescriber	464	11.7
Pharmacist did not dispense medication	258	6.5
Patient counselling and education provided	580	14.7
**Total**	**3955**	**100.0**
**Outcomes of medication error(s) interventions**		
Potential/actual medication error(s) resolved	3208	97.4
Potential/actual medication error(s) NOT resolved	84	2.6
**Total**	**3292**	**100.0**

* Some patients had ≥ 1 medication errors; some medication errors required ≥ 1 interventions; and outcomes of all interventions were not indicated.

## Discussion

The incidence rate of medication errors in this setting was high following the active screening program. This is somewhat consistent with the expectations from previous research findings [2]–[5]. In HIV treatment setting, there is increased likelihood for polypharmacy, durations of therapy is life-long, and there may be high patient burden and inadequate trained health workforce. These factors may increase the risk of occurrence of medication errors [2]–[5], [7]–[9]. The incidence rate of medication errors was higher among patients aged ≥15years old compared to those aged < 15 years; however the difference was not statistically significant. This is consistent with the age distribution of the participants as majority (over 90%) was aged ≥15years old. Despite the training of health workers (doctors, pharmacists and nurses) on the use of antiretroviral drugs for HIV prevention and treatment based on the provisions of the national HIV treatment guidelines [12], the incidence rate of medication errors in this setting was high. The high detection rate of medication errors may be attributed to the training intervention provided to the pharmacists, and that may have improved their knowledge and skills to identify and resolve medication errors in HIV treatment setting.

The major medications errors identified were related to prescription of incorrect ART regimens, potential drug-drug interaction or contraindication and inappropriate duration and/or frequency of medication. This is consistent with previous research findings [6], [7]. Antiretroviral therapy requires the use of triple combination of antiretroviral drugs (ARVs) as recommended by the Nigerian national treatment guidelines [12]. Drug interactions may occur due to inappropriate combination of ARVs or use of concomitant medicines in cases of co-morbidities. These drug combinations need to be carefully selected to ensure patient safety and rational use of these medicines. Only patients who are eligible as recommended by the treatment guidelines [12] are commenced on life-long ART. The antiretroviral drugs regimens are also used for post-exposure prophylaxis and the prevention of mother-to-child transmission of HIV. For the purpose of prophylaxis, ARVs are given for a specified duration as recommended by the treatment guideline [12]. The cases of inappropriate duration of medication may be related to patients receiving ARVs for prophylaxis; and whereas inappropriate frequency of dosing may be related to patients on either life-long ART or those receiving prophylaxis.

Almost all participants who had medication error(s) were provided one kind of pharmacist’s intervention or the other; and these interventions included mainly the contacting of the prescriber for clarification or resolution of errors.

The knowledge of incidence rate and the types of medication errors identified among HIV-infected patients receiving antiretroviral drugs will be very useful when planning and designing targeted interventions aimed at improving the quality of health care systems in resource-limited settings. The study demonstrated that active screening for medication errors is feasible in resource-limited settings following a capacity building intervention. However, inadequate pharmacy personnel and high patient load were the major limitations to achieving active screening for medication errors, thus some medication errors in patients may have been missed. The study reported medication errors identified by pharmacist, thus it is likely that dispensing errors committed by the pharmacists may be underestimated while the errors resulting from the activities of the patients and prescribers may be overestimated. This may bias the overall measure of effects in this study. Other potential limitations of this study include lack of inclusion of all treatment sites due to lack of medication errors screening programs uniformly, and potential for pharmacists not to report some medication errors due to fear of severing the interpersonal relationship with other health workers or other unknown concerns. These factors may overestimate or underestimate the study findings.

## Conclusion

The incidence rate of medication errors was somewhat high; and majority of identified errors were related to prescription of incorrect ART regimens and potential drug-drug interactions; the prescriber was contacted and the errors were resolved in majority of cases. The study demonstrated that active screening for medication errors is feasible in resource-limited settings following a capacity building intervention.

## References

[1] NCCMERP (2013) About Medication Errors [online] Available: http://www.nccmerp.org/aboutMedErrors.html.Accessed 4 February 2013.

[2] Schoen C, Osborn R, How SK, Doty MM, Peugh J (2008) ‘In chronic condition: experiences of patients with complex health care needs, In eight countries, 2008 - Chronically ill U.S. patients have the most negative access, coordination, and safety experiences’, *Health Affairs*, vol. 28, no. 1, w1–w16. 10.1377/hlthaff.28.1.w1 19008253

[3] McGlynn E, Asch S, Adams J, Keesey J, Hicks J et al (2003) ‘The quality of health care delivered to adults in the United States’, *The New England Journal of Medicine*, vol. 348, pp. 2635 – 2645, 10.1056/NEJMsa022615 Accessed 4 February 2013. 12826639

[4] Ahuja N, Zhao W, Xiang H (2012) Medical errors in US paediatric in-patients with chronic conditions, *Paediatrics*, October: vol. 130 (4) , pp 786 – 793 10.1542/peds.20112555 Accessed 8^th^ June 2013. 22966036

[5] Cornish PL, Knowles SR, Marchesano R, Tam V, Shadowitz S et al (2005) ‘Unintended medication discrepancies at the time of hospital admission’ *Archives of Internal Medicine*, vol. 165(4), pp. 424–429. 10.1001/archinte.165.4.424 15738372

[6] Yehia BR, Mehta JM, Ciuffetelli D, Moore RD, Pham PA et al (2012) ‘Antiretroviral medication errors remain high but are quickly corrected among hospitalized HIV-infected adults’, *Clinical Infectious Diseases*, vol. 55(4) , pp. 593 – 599, 10.1093/cid/cis491 Accessed 5^th^ June 2013. PMC3520383 22610923

[7] Rastegar DA, Knight AM, Monolakis JS (2006) ‘Antiretroviral medication errors among hospitalized patients with HIV infection’, *Clinical Infectious Diseases*, vol. 43(7), pp. 933 – 938. Accessed 5^th^ June 2013. 10.1086/507538 16941379

[8] Rao N, Patel V, Grigoriu A, Kaushik P, Brizuela M (2012) Antiretroviral therapy prescribing in hospitalized HIV clinic patients, HIV Medicine, vol. 13 (6), pp. 367 -371, 10.1111/j.1468-1293.2011.00977 Accessed 5^th^ June 2013. 22252216

[9] Faragon JJ (2006) Antiretroviral medication errors in patients with HIV infection, *Adv Stud Pharm*, vol 3(5), pp.: 179 – 186.

[10] PLAN – Health (2011) *Nigeria Hosts First National Conference on Human Resources for Health with PLAN – Health Support*. Human Resources for Health National Conference. October 2011. Abuja: PLAN – Health.

[11] Uneke C, Ogbonna A, Ezeoha A, Oyibo P, Onwe F et al (2008) Innovative Health Research Group: The Nigeria health sector and human resource challenges. *The Internet Journal of Health*, vol 8(1), DOI: 10.5580/d5a Accessed 10^th^ June 2013.

[12] Federal Ministry of Health, FMOH (2010) “National Guidelines for HIV/AIDS Treatment and Care in Adolescents and Adults, Federal Ministry of Health Abuja, Nigeria,” October 2010.

